# Sample storage conditions induce post-collection biases in microbiome profiles

**DOI:** 10.1186/s12866-018-1359-5

**Published:** 2018-12-27

**Authors:** Samir V. Jenkins, Kieng B. Vang, Allen Gies, Robert J. Griffin, Se-Ran Jun, Intawat Nookaew, Ruud P. M. Dings

**Affiliations:** 1 Department of Radiation Oncology, University of Arkansas for Medical Sciences, Little Rock, AR USA; 2 Center for Integrative Nanotechnology Sciences, University of Arkansas, Little Rock, AR USA; 3 Department of Microbiology and Immunology, University of Arkansas for Medical Sciences, Little Rock, AR USA; 4 Department of Biomedical Informatics, University of Arkansas for Medical Sciences, Little Rock, AR USA

**Keywords:** Gut microbiome, 16S rRNA, Alpha diversity, Fecal microbiome transplantations, Metabolic function analysis

## Abstract

**Background:**

Here we investigated the influence of different stabilization and storage strategies on the quality and composition of the fecal microbial community. Namely, same-day isolated murine DNA was compared to samples stored for 1 month in air at ambient temperature, with or without preservative buffers (i.e. EDTA and lysis buffer), different temperatures (i.e. 4 °C, − 20 °C, and − 80 °C), and hypoxic conditions.

**Results:**

Only storage in lysis buffer significantly reduced DNA content, yet without integrity loss. Storage in EDTA affected alpha diversity the most, which was also reflected in cluster separation. Distinct changes were also seen in the phyla and bacterial species abundance per storage strategy. Metabolic function analysis showed 22 pathways not significantly affected by storage conditions, whereas the tyrosine metabolism pathway was significantly changed in all strategies except by EDTA.

**Conclusion:**

Each long-term storage strategy introduced a unique post-collection bias, which is important to take into account when interpreting data.

**Electronic supplementary material:**

The online version of this article (10.1186/s12866-018-1359-5) contains supplementary material, which is available to authorized users.

## Background

Commensal bacteria reside in many parts of the human body, including the oral cavity, respiratory tract, urogenital tract, skin and maybe most notably the gastro-intestinal tract [1, 2]. In the human intestine alone, the total number of bacteria is estimated to be around 1 × 10^14^ (~ 2 kg), outnumbering the eukaryotic cells by a factor of ten or more [1, 3, 4]. A disturbance in the composition of the microbiota, also termed dysbiosis, may result in an increase in the risk of various diseases, including inflammatory (e.g., inflammatory bowel disease, Crohn’s disease, and colon cancer), autoimmune (e.g., celiac disease, arthritis, and multiple sclerosis), allergy-based (e.g., asthma and atopy), metabolic (e.g., diabetes, obesity, metabolic syndrome, and kwashiorkor), and psychological/neurological (e.g., autism) diseases [1, 2, 5]. Moreover, it is becoming increasingly clear that the microbiome is not only integral to providing significant insight into disease states, but it can also be applied or manipulated in a therapeutic fashion. For example, clinically, fecal microbiome transplantation (FMT) of healthy donors is a successful treatment for *Clostridium difficile* infection [6].

The most common method to identify commensal bacteria is through next generation sequencing, with 16S ribosomal RNA (rRNA) being widely used [7]. 16S sequencing of fecal matter is an effective, non-invasive method to assess the gut microbiome, as it generates significant taxonomic information often to the level of bacterial species. Unfortunately, this sensitivity also means that sample handling can affect the results and introduce a bias within the generated profile.

Thus far, studies have analyzed changes in the bacterial population as a result of different commercially available isolation kits [8, 9] or different storage conditions over a relatively short period of time. For example, storage at ambient room temperature (RT; ~ 20 **°**C) for 2 days introduced differences in the population [7, 10]. Similarly, short-term storage in different media, such as lysis buffers or nucleic acid stabilizers (e.g. Ethylenediaminetetraacetic acid (EDTA)) have also been reported to significantly change the microbiome profile [11, 12]. These reports typically use a storage time of one or 2 days as it represents a clinical timescale between a patient providing a sample and the initial analysis. However, basic and translational sciences often rely on longitudinal animal models resulting in multiple samples per individual over time as the disease state progresses. Additionally, in both clinical and academic settings it is often advantageous to work-up many samples at once, rather than immediately upon collection of individual samples. As a result samples are collected and stored for extended periods of time prior to analysis.

Aside from the short-term influences of the various temperatures and stabilization buffers on the microbiome profile, samples are almost always stored under normoxic conditions (~ 21% O_2_). This has the potential, at least in theory, of introducing a false positive bias towards aerobic bacteria during long-term storage.

Here, we investigated the effects of storing fecal samples long-term in a variety of storage conditions (Table 1). The fecal matter of C57BL/6 mice was used, as they are one of the most common animals employed in research, especially in regards to studying gut microbiota related diseases [13]. The bacterial DNA of the same day isolated samples assessed by 16S rRNA amplicon sequencing was compared to 1 month of storage at RT, with or without preservative buffers (i.e. 100 mM EDTA and lysis buffer), different temperatures (i.e. 4 **°**C, − 20 **°**C, and − 80 **°**C), and hypoxic conditions (i.e. < 10 mmHg pO_2_).

**Table 1:** Sample stabilization and storage conditions

	Classification	Time	Solution	Temperature	Oxygen level^*a*^
1.	Fresh	< 1 days	NA	NA	NA
2.	EDTA	33 days	100 mM EDTA	20 °C	Ambient
3.	Lysis	33 days	S1 and S2^*b*^	20 °C	Ambient
4.	RT	33 days	NA	20 °C	Ambient
5.	4 °C	33 days	NA	4 °C	Ambient
6.	−20 °C	33 days	NA	−20 °C	Ambient
7.	−80 °C	33 days	NA	−80 °C	Ambient
8.	Hypoxia	33 days	NA	20 °C	< 10 mmHg pO_2_

^a^Ambient pO2 is anticipated to be 160 mmHg pO_2_ ≅ 21% O_2_; 10 mmHg pO_2_ ≅ 1.3% O_2_

^b^Proprietary. Obtained from PureLink Microbiome DNA Purification Kit (Invitrogen #A29790). NA = Not Applicable

## Methods

### Fecal collection and storage conditions

Experiments were approved by the University of Arkansas for Medical Sciences Institutional Animal Care and Use Committee and performed in accordance with relevant regulations and guidelines. Pooled fecal matter of healthy 12 week old female C57BL/6 mice (JAX #000664, The Jackson Laboratory; *n* = 5) was collected and apportioned. Individual pellets were collected, pooled, and then apportioned at 200 mg per sample and randomly distributed over the different 8 conditions (Table 1): same day isolation, or stored for 33 days at room temperature (RT), 100 mM EDTA at RT (in 5 ml 10 mM Tris-HCL pH 8.0), lysis buffer at RT (Invitrogen S1 and S2 solution, #A29790), refrigerated at 4 °C, frozen at − 20 °C or − 80 °C or stored under hypoxic conditions (< 10 mmHg pO_2_) at RT. All samples were stored in the dark, and homogenized immediately prior to DNA extraction.

Hypoxic conditions were created by storing the sample in purged airtight glass syringes (Restek 2.5MDR-VLL-GT) in an anaerobe chamber (Forma Scientific Inc.) with a gas mix (5% H_2_, 5% CO_2_ and 90% N_2_) generating hypoxic conditions, as defined as oxygen concentrations below 10 mmHg pO_2_ or 1% O_2_ [14, 15].

### DNA extraction and quantification

After the long-term storage strategies DNA extraction was performed using PureLink™ Microbiome DNA Purification Kit (#A29790; Invitrogen, Carlsbad, CA) according to the manufacturer’s instruction. For each storage condition duplicate samples were stored under identical conditions but in separate containers until processing. Briefly, samples were suspended in lysis buffer and heated to 60 °C for 15 min prior to 15 min of horizontal vortexing to homogenize the samples. Samples were centrifuged and the supernatant collected. From this several processing steps were performed to remove residual protein and the final DNA sample was eluted in 100 μL of nuclease free H_2_O. The concentration and purity were determined by the A_260_/A_280_ value (Cytation 5; BioTek, Winooski VT, USA).

### 16S rRNA gene sequencing

The V3 and V4 hypervariable regions of the bacterial 16S rRNA gene were amplified using primers containing Illumina adapters following Illumina’s 16S Metagenomics sequencing Library Preparation Protocol (# 15044223, Rev. B) optimized for the Illumina MiSeq system. In brief, Kapa Library Amplification Kit (# KK2611) was used for polymerase chain reaction (PCR) and products were cleaned using Beckman Coulter Agencourt AMPure XP Beads (# A63881) according to the 16S Metagenomics protocol. The full-length primer sequences used described by Klindworth et al., [16] in standard IUPAC nucleotide nomenclature are:

16S Amplicon PCR Forward Primer = 5'

CG TCG GCA GCG TCA GAT GTG TAT AAG AGA CAG CCT ACG GGA GGC AGC AG

16S Amplicon PCR Reverse Primer = 5'

GTC TCG TGG GCT CGG AGA TGT GTA TAA GAG ACA GGA CTA CCA GGG TAT CTA AT

The resulting products were quantified and 5 μl of each sample was then used for indexing using Illumina’s Nextera Dual Indexing Strategy (Additional file 1: Table S1). Subsequently, the indexed samples were cleaned, quantified, and checked for size on an Agilent Tapestation 2200 using D1000 Tapes (# 5067–5582) and associated reagents (# 5067–5583). All samples were found to be in the expected size range (600–650 bp) (Additional file 1: Figure S1) and their concentrations were adjusted to 4 μM and prepared for loading on the Illumina MiSeq according to Illumina’s 16S Metagenomics Protocol. Subsequently they were denatured and loaded on the Illumina MiSeq at 8 pM and sequenced paired-end (2 × 300) using a MiSeq® Reagent Kit v3 (600 cycle) (# MS-102-3003).

### Data processing and analysis

After demultiplexing the raw reads into individual samples BBmap software was used for preprocessing. Subsequently quality trimming was performed using BBDuk (v.37.02) with a quality score cut-off 15 [17]. The high quality pair-end reads were then merged by BBMerge with an overlap length cut-off region of 22 bases [17]. Reads shorter than 200 bp were discarded and were used in OTU picking step. The OTU picking was performed by QIIME software (v.1.9.1) with open OTU picking workflow using the default option [18]. The results from OTU picking step were imported to the R suite environment through PhyloSeq packages for statistical analysis and illustration of results [19]. The metabolic capability projection from the detected OTUs was derived from functional profiles inferred by the PanFP method, which can be applied to any OTU picking protocols [20]. The two-tailed Student’s t-test and the non-parametric Mann-Whitney were used to determine the validity of differences between the data sets, presented as the mean ± standard errors of the mean. A *P* value of 0.05 or less was considered significant.

## Results

### Long-term storage in lysis buffer reduces recoverable DNA, without loss of DNA integrity

**Fig. 1:**
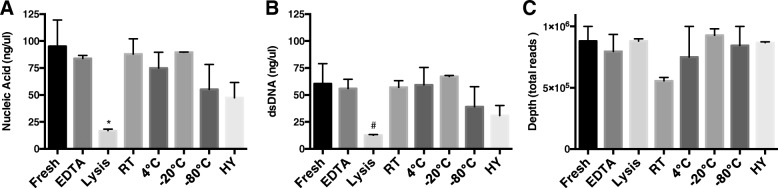
Recoverable DNA from fecal matter under 8 different storage conditions. **a**. Total nucleic acid content recovered from fecal matter based on the absorbance at 260 nm. **b**. Double stranded DNA (dsDNA) concentrations recovered from fecal matter based on the fluorescent absorbance of the Qubit dsDNA broad range (BR) assay reagent. **c**. Bacterial DNA sequencing coverage achieved per different storage condition. The data is presented as the mean ± SEM. *p < 0.05, ^#^*p* = 0.06

For each storage condition the DNA isolation efficiency was compared to the yield from samples immediately isolated (Fig. 1). We found based on the absorbance at 260 nm that storing the samples in lysis buffer resulted in the lowest nucleic acid yield: in the fresh samples we recovered 95 ± 25 ng/μl nucleic acid, whereas storage in the lysis buffer reduced this to 16.9 ± 1.5 ng/μl (Fig. 1a). None of the other storage conditions caused a significant drop in nucleic acid yield. These trends were also seen in the double stranded DNA (dsDNA) yields. Whereas fresh samples resulted in an average of 60.4 ± 18.6 ng/μl dsDNA, storage in lysis buffer reduced this to 12.7 ± 0.6 ng/μl (Fig. 1b). Although the lysis buffer reduced both the nucleic acid and the dsDNA content, the integrity was maintained. Namely based on the Taqman analysis (Additional file 1: Figure S1) and the total reads, storing the sample in lysis buffer did not interfere with the quality of the bacterial DNA. Storing at RT (~ 20 °C), however, did reduce the integrity. Whereas fresh isolations rendered on average 8.8 × 10^5^ total reads, storing the sample at RT provided only 5.6 × 10^5^ total reads on average, a reduction of almost 40% (*p* < 0.05; Fig. 1c). The other storage conditions did not alter the number of reads significantly.

### Sample storage in EDTA reduces the alpha diversity

**Fig. 2:**
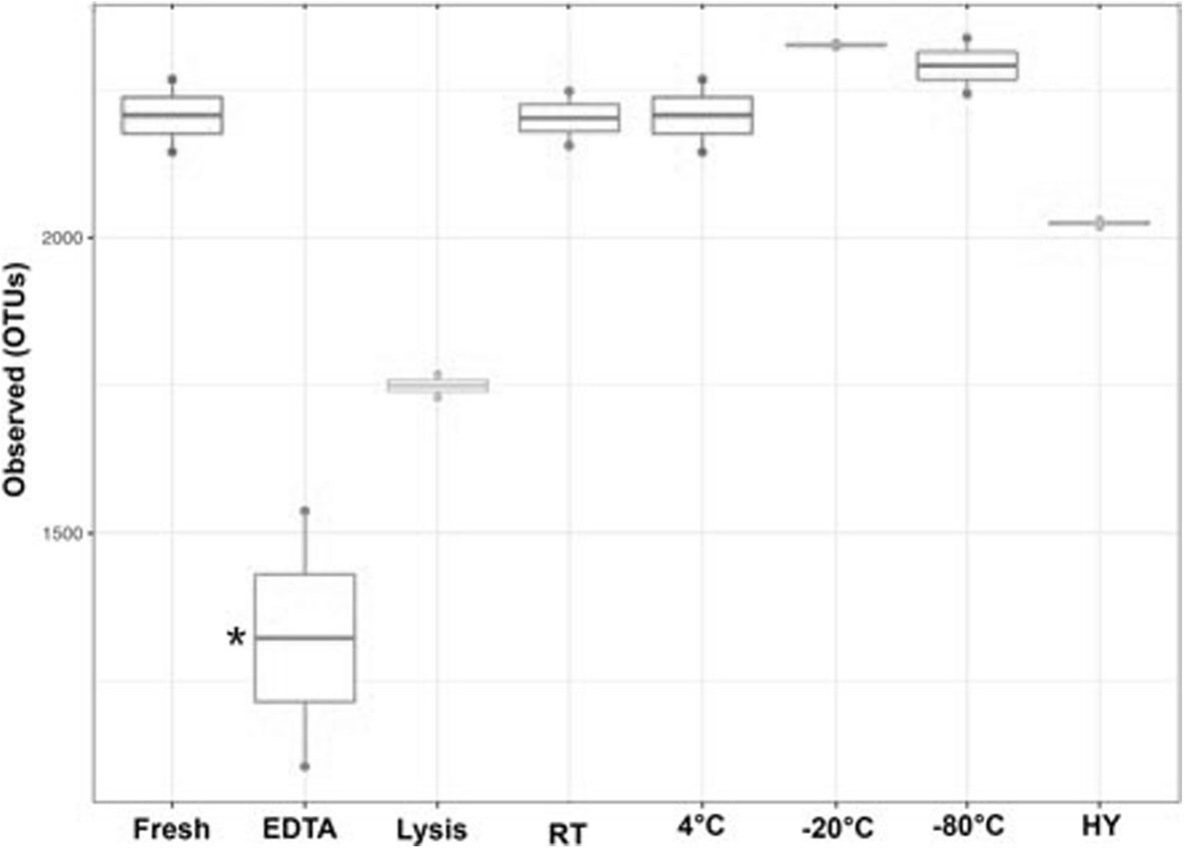
The observed unique operational taxonomic units (OTUs) between storage conditions and individual samples. The boxplots show the alpha diversity as median, quartile, smallest and largest observations (circles). *p < 0.05

In order to assess whether storage condition could alter the alpha diversity, the variety within a sample, we quantified the amount of observed operational taxonomic units (OTUs) richness (Fig. 2). Of the different storage conditions, lysis buffer caused a modest drop in observed OTUs from 2205 OTUs to 1750 OTUs as compared to immediate isolations of fresh samples. Long-term storage of the samples in EDTA, however, caused a significant drop in OTUs down to 1321 OTUs (*p* < 0.05). Keeping the samples for an extended time at different temperatures did not significantly change the observed OTUs, nor did storing the under hypoxic conditions. We also analyzed the alpha diversity by the Chao1 estimator and found the same trends as seen for the observed OTUs (Additional file 1: Figure S2).

### Microbiota ordination and taxon stored in lysis buffer clusters with fresh isolated samples

**Fig. 3:**
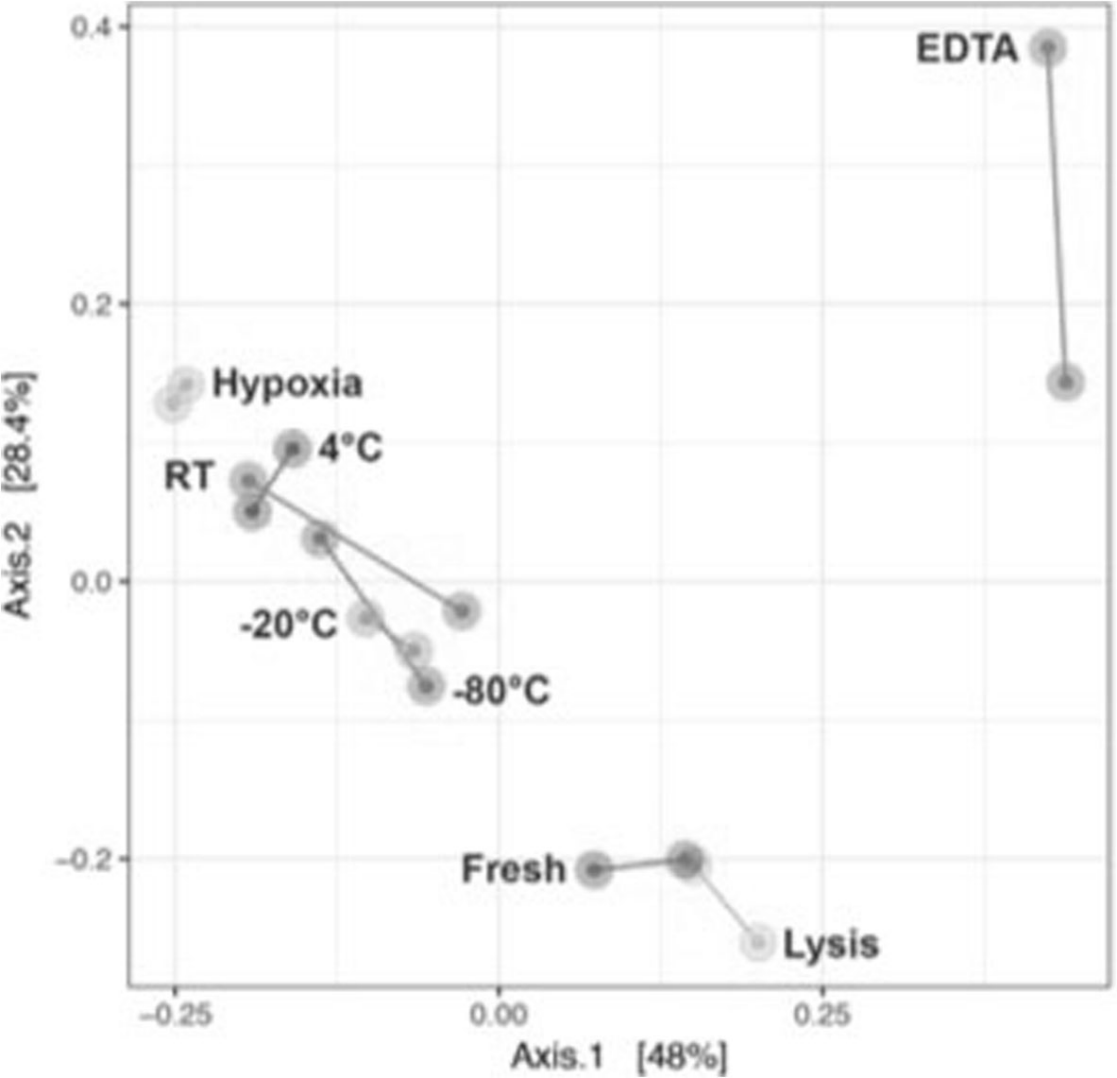
Clustering of samples by principal coordinate analysis (PCoA). PCoA based on Bray-Curtis dissimilarity. The first two principle coordinates are plotted on the x- and y-axis, respectively. This represents 76.4% of the total variation

Principal coordinate analysis (PCoA) of Bray Curtis distances revealed that the different storage conditions caused 3 clusters (Fig. 3). Notably the fresh isolated bacterial DNA clustered best with the DNA isolated from samples stored with the lysis buffer. The samples stored at different temperature ranging from − 80 **°**C to 20 **°**C also clustered together. Anaerobic stored samples clustered by themselves and samples stored in EDTA changed the most and were separated from the other clusters (Fig. 3).

### Hypoxic storage conditions induces the greatest significant changes in relative Phyla abundance

**Fig. 4:**
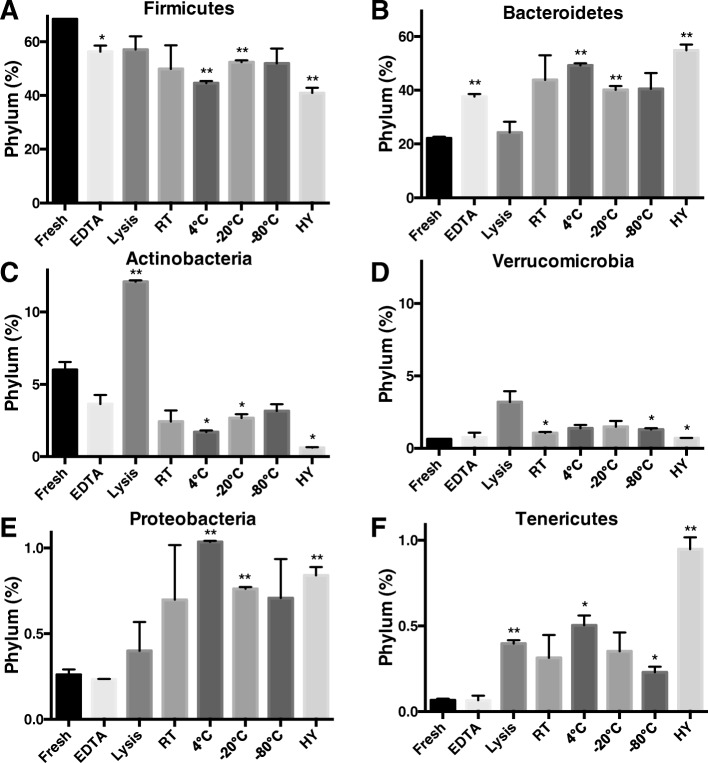
Phylum composition diversity following incubation under 8 different storage conditions. The relative abundance of (**a**) Firmicutes, (**b**) Bacteroidetes, (**c**) Actinobacteria, (**d**) Verrucomicrobia, (**e**) Proteobacteria and (**d**) Tenericutes as a function of storage condition. *p < 0.05, **p < 0.01. The data is presented as the mean ± SEM

Relative phyla abundance analysis identified Firmicutes (68%) and Bacteroidetes (22%) as the two dominant phyla in the freshly isolated samples (Table 2 and Fig. 4). This relative abundance of the Firmicutes was reduced by all storage conditions to various degrees, with the greatest relative reduction of − 27.5% by hypoxic conditions (*p* < 0.05). Universally this relative abundance reduction in Firmicutes was associated with an increase in Bacteroidetes with the greatest increase while stored under hypoxic conditions (+ 32.7%; *p* < 0.05). Whereas all storage conditions reduced the relative abundance of Actinobacteria, storage in lysis buffer caused a doubling of relative abundance from 6 to 12% (*p* < 0.05). Overall, as compared to freshly isolated samples, storage under hypoxic conditions was the only strategy that caused significant changes in the relative abundance of all phyla (Table 2 and Fig. 4).

**Table 2:** Mean percent difference in the relative phyla abundance in different storage conditions. Data is presented as mean ± SEM. Significant differences are highlighted in bold (*p* < 0.05)

Phylum	Fresh	EDTA vs Fresh	Lysis vs Fresh	RT vs Fresh	4 °C vs Fresh	− 20 °C vs Fresh	− 80 °C vs Fresh	HY vs Fresh
Firmicutes	68.42% ± 0.06%	− 12.17% ± 2.33%	−11.36% ± 4.97%	−18.57% ± 8.89%	−23.85% ± 0.87%	− 16.07% ± 0.73%	− 16.56% ± 5.64%	− 27.52% ± 2.02%
Bacteroides	22.14% ± 0.59%	+ 15.52% ± 0.96%	+ 2.14% ± 3.98%	+ 21.72% ± 9.20%	+ 27.06% ± 0.79%	+ 18.01% ± 1.38%	+ 18.38% ± 5.91%	+ 32.7% ± 2.17%
Actinobacteria	6.00% ± 0.54%	− 2.36% ± 0.65%	6.08% ± 0.11%	− 3.58% ± 0.77%	−4.29% ± 0.11%	−3.33% ± 0.27%	−2.84% ± 0.46%	−5.38% ± 0.04%
Verrucomicrobia	0.63% ± 0.01%	+ 0.13% ± 0.31%	+ 2.56% ± 0.74%	+ 0.42% ± 0.08%	+ 0.74% ± 0.25%	+ 0.85% ± 0.41%	+ 0.66% ± 0.11%	+ 0.08% ± 0.01%
Proteobacteria	0.26% ± 0.03%	−0.03% ± 0.01%	0.14% ± 0.17%	+ 0.44% ±0.32%	+ 0.78% ± 0.01%	+ 0.50% ± 0.01%	+ 0.45% ± 0.23%	+ 0.58% ± 0.05%
Tenericutes	0.07% ± 0.01%	+ 0.01% ± 0.03%	+ 0.33% ± 0.02%	+ 0.25% ± 0.13%	+ 0.44% ± 0.06%	+ 0.29% ± 0.11%	+ 0.16% ± 0.03%	+ 0.88% ± 0.07%

### Metabolic function analysis

The pangenome-based functional profiles (PanFP) method was used to infer functional profiles and the changes induced by the storage conditions. The analysis was performed based on 5110 genes grouped in 276 associated pathways (Table 3 and Additional file 1). Every individual long-term storage condition had some degree of modulation on a genomic as well as pathway level as compared to fresh samples. On a genomic level, storing under hypoxic conditions or at 4 °C induced the most significant changes in relative gene expression (~ 65 - 69%, Table 3). Storing at − 20 °C caused significant changes in approximately 52% of the genes, whereas storing the samples in lysis buffer, EDTA, RT, or − 80 °C caused the fewest significant changes in relative gene expression namely ~ 14 - 21%. Pathway analysis showed similar trends. Long-term storage under hypoxic condition, or at 4 °C and − 20 °C caused the most significant changes (~ 64 - 72%); EDTA, lysis buffer, RT and − 80 °C cause fewer changes (~ 10 - 18%). None of the pathways were affected by all of the storage strategies. However, the tyrosine metabolism pathway (KEGG pathway ko00350) was most often affected by different conditions: 6 out of the 7 storage strategies affected this pathway significantly. Storage in EDTA, however, did not increase or suppress its relative expression as compared to immediately isolated. Nearly 8% (22 pathways) were not significantly affected by any long-term storage strategy (Table 3 and Additional file 1). Further analysis revealed that some storage conditions saw enhancement of pathways as their greatest relative change, i.e. apoptosis (ko04214) for 4 °C storage (*p* < 0.00001), fatty acid biosynthesis (ko00061) for hypoxic conditions (*p* < 0.0003) and toluene degradation (ko00623) for EDTA storage (*p* < 0.0003) and for lysis buffer (*p* < 0.0006).

**Table 3:** Functional metabolic genes and pathways affected per storage condition as compared to fresh samples

Condition	Genes^a^		Pathways^b^				
	Number	%	Number	%	Greatest change	Log2FC	*p* value
Fresh	5110	100	276	100	NA	NA	NA
EDTA	978	19.1	27	9.8	Toluene degradation	+ 3.9	< 0.0003
Lysis	689	13.5	40	14.5	Toluene degradation	+ 4.5	< 0.0006
RT	1050	20.5	40	14.5	Lysine degradation	−2.1	< 0.003
4 °C	3533	69.1	198	71.7	Apoptosis	+ 3.2	< 0.00001
−20 °C	2637	51.6	183	66.3	Alzheimer Disease	−1.9	< 0.0006
−80 °C	1088	21.3	50	18.1	Beta-lactam resistance	−2.0	< 0.005
HY	3317	64.9	176	63.8	Fatty acid biosynthesis	+ 5.3	< 0.0003
Pathways not affected			22	8.0	NA	NA	NA
Affected by all conditions			0	0	none	NA	NA
Pathway affected by 6 out of 7 conditions (excludes EDTA)			1	0.4	Tyrosine metabolism	varies	varies

^a^5110 genes and ^b^276 pathways were annotated. NA = Not Applicable

The other storage conditions induced a suppression of pathways as their greatest relative changes, i.e. Alzheimer disease (ko05010) for − 20 °C (*p* < 0.0006), lysine degradation (ko00310) for RT (*p* < 0.003) and beta-lactam resistance (ko01501) for − 80 °C (*p* < 0.005).

## Discussion

Sequencing-based assessment of the fecal microbiome has become increasingly important in science and clinical practice as more correlations and causal relationships are being identified between disease states and microbiome profiles. Therefore it is crucial that no post-collection bias is introduced during long-term sample storage as this might mask proper clinical diagnosis. Here we investigated the influence of different stabilization and storage strategies, i.e. different preservative buffers, temperatures, and oxygen concentrations, on the quality and composition of murine fecal microbiome after being stored for over 1 month.

Interestingly, we found that storage in commercially available lysis buffer reduced the DNA content the most, yet did not cause loss of DNA integrity. Although DNA binding to the plastic microfuge tubes could be a possible explanation, this is unlikely as all samples were stored in the commercially provided receptacles and moreover homogenization by bead-beating was performed just prior to the DNA isolation. It is more likely a function of the storage in a liquid medium causing non-specific DNA degradation. However, this degradation was not observed with the samples stored in EDTA, as EDTA inhibits DNA degradation. All other storage conditions did not induce significant changes in DNA content or integrity. It must be noted that the proprietary lysis buffer was not optimized for long-term storage purposes. Many studies have historically stored their samples in EDTA to inhibit the enzymatic action of DNase [11]. We found that long-term storage in EDTA affected the alpha diversity the most, which was also reflected in cluster separation as determined by PCoA.

The overall bacterial taxonomic groups found in murine fecal matter were similar to previous findings, with Firmicutes and Bacteroidetes being the two major phyla [21–23].

Until now this difference has been mostly attributed to differences in species, mouse strains, mouse vendors, or diet [23]. Here we uncovered a new potential confounder, namely storage condition, as this can also significantly influence the measured abundance and composition of the microbiome. We found that the dominance of Firmicutes over Bacteroidetes is minimized over long-term storage or even reversed when stored at 4 °C or under hypoxic conditions. It is unclear whether this is due to the relative enhanced DNA degradation of the Bacteriodetes or because of a growth-related increase of Firmicutes. This post-collection augmentation is crucial to take into account while interpreting data and moreover regarding fecal microbiome transplantations (FMT), which have been increasingly adapted into clinical practice [6].

On the other hand, this change can also be used to one’s advantage when certain phyla or bacterial strains are preferred. For instance, in the past *Escherichia coli* was falsely interpreted as the primary gut microbe due to the ease with which it could be cultured and detected [24]. Extended storage in relatively oxygen rich conditions favored the growth of (facultative) aerobes, which ultimately affected the measured population distribution and misrepresented the importance and significance of anaerobes. Here we found that storage under hypoxic conditions favors bacterial strains within the Bacteroidetes and Proteobacteria phyla as their relative abundance increased without significant loss in DNA content or integrity. Thus one might consider long-term storage under low oxygen tension when there is an interest in an in-depth analysis of facultative or obligate anaerobe bacteria commonly found within Bacteroidetes and Proteobacteria phyla. Hypoxic conditions also induced changes in regard to metabolic function on a genomic as well as pathway level. The greatest change was observed within the enhancement of the fatty acid biosynthesis pathway- an anaerobic pathway for synthesizing unsaturated fatty acids, possibly due to the increase in Tenericutes [25].

PanFP, a metagenome inference software, allows for functional profiling of the 16S-based microbiome, generating a rudimentary prediction of the metabolic capacity of the microbiome. Here, we examined whether there were any general changes regarding the metabolic capacity of the microbiome in response to storage conditions. PanFP analysis indicated that the common method of storage at − 80 °C caused a relative change in more than 21% of the genes and approximately 18% of the associated pathways, as compared to the immediately isolated samples. Storage in preservation and stabilization buffers induced fewer significant changes as compared to immediate isolation of fresh samples. However these conditions came at the cost of a significant reduction in DNA content for lysis buffer or a reduction in OTUs, cluster separation and significant changes in phylum distribution for EDTA. Intriguingly, storage at − 20 °C had more than twice the genomic changes and more than thrice the metabolic pathways changes as compared to − 80 °C (Table 3). Moreover, long-term storage at 4 °C caused the most modulations, on both the genomic as well as pathway level, in almost two-third of the cases. Thus taken together the coldest condition, − 80 °C, is preferred when immediate isolation is not possible or practical.

From a pathway perspective 8% of the pathways were not affected by any of the investigated storage conditions. However the genes associated with the tyrosine metabolism pathway were affected in 6 out of 7 conditions. Thus tyrosine metabolism investigations would be preferred on fresh samples only (Table 3). Although PanFP was developed and optimized for 16S amplicon sequence data [20], further profiling by e.g. whole-genome shotgun metagenomic sequencing would strengthen these predictions.

## Conclusions

All 7 different strategies introduced a unique post-collection bias. Thus it is important to take this into account during the data interpretation of past, current, or future microbiome profile studies, as well as during therapeutic approaches involving stool-derived treatments (e.g. FMT). Alternatively, this post-collection bias can be used to an advantage if one favors a particular sub-set of phyla or bacteria (e.g. anaerobes), which can be relatively enriched during long-term storage.

## Additional file

Additional file 1: **Table S1.** The indexing scheme. Datasheet metabolic function gene and pathway analysis. **Figure S1.** Tapestation 2200 images using D1000 Tapes and D1000 reagents. a, Indexed samples were found to be in the expected size range (600-650 bp). B, Sizes corresponding to the bands detected in the ladder in the first lane of panel A. **Figure S2.** Detected alpha diversity in fecal matter following incubation under 8 different storage conditions. Chao1 richness estimator between storage conditions and individual samples. (DOCX 438 kb)

## Abbreviations

dsDNA: Double stranded DNA
EDTA: Ethylenediaminetetraacetic acid
FMT: Fecal microbiome transplantation
HY: Hypoxia
OTU: Operational taxonomic units
PCoA: Principal coordinate analysis
RT: Room temperature
