# Supplementary material for: Sample storage conditions induce post-collection biases in microbiome profiles
Source: BMC Microbiol. 2018 Dec 27;18:227. doi: 10.1186/s12866-018-1359-5 (PMC6307155; doi:10.1186/s12866-018-1359-5)
Supplement: Supplementary file 1 — Table S1. The indexing scheme. Datasheet metabolic function gene and pathway analysis. Figure S1. Tapestation 2200 images using D1000 Tapes and D1000 reagents. a, Indexed samples were found to be in the expected size range (600-650 bp). B, Sizes corresponding to the bands detected in the ladder in the first lane of panel A. Figure S2. Detected alpha diversity in fecal matter following incubation under 8 different storage conditions. Chao1 richness estimator between storage conditions and individual samples. (DOCX 438 kb) [file 12866_2018_1359_MOESM1_ESM.docx]

**Additional file 1**

**Table S1,** The indexing scheme.

| **Sample** | **Condition** | **i7 Sequence** | **i5 Sequence** |
| --- | --- | --- | --- |
| **1** | Fresh | TAAGGCGA | CTCTCTAT |
| **2** | Fresh | CGTACTAG | TATCCTCT |
| **3** | RT | AGGCAGAA | AGAGTAGA |
| **4** | RT | TCCTGAGC | GTAAGGAG |
| **5** | Lysis | GGACTCCT | ACTGCATA |
| **6** | Lysis | TAGGCATG | AAGGAGTA |
| **7** | EDTA | GCTACGCT | CTCTCTAT |
| **8** | EDTA | CGAGGCTG | TATCCTCT |
| **9** | 4C | AAGAGGCA | AGAGTAGA |
| **10** | 4C | GTAGAGGA | GTAAGGAG |
| **11** | -20C | TAAGGCGA | ACTGCATA |
| **12** | -20C | CGTACTAG | AAGGAGTA |
| **13** | -80C | AGGCAGAA | CTAAGCCT |
| **14** | -80C | TCCTGAGC | GCGTAAGA |
| **15** | HY | CTCTCTAC | CTAAGCCT |
| **16** | HY | CAGAGAGG | GCGTAAGA |


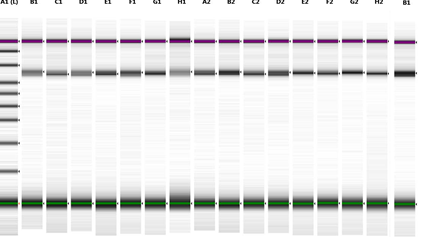


**B**

**A**


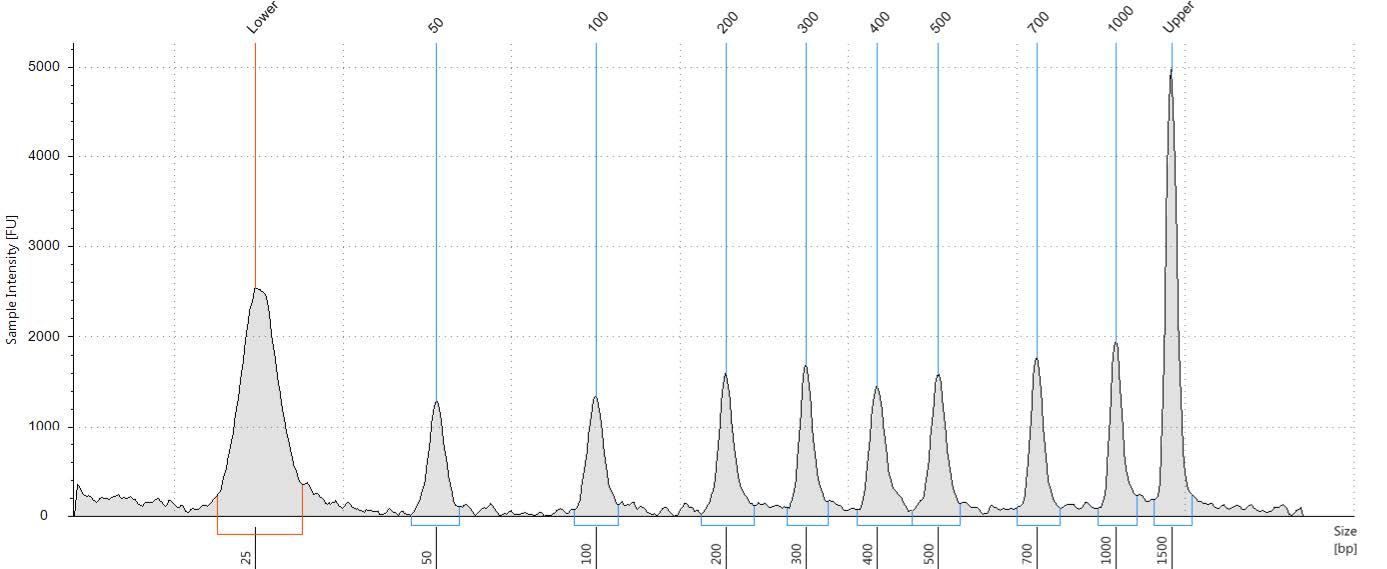


**Figure S1**, Tapestation 2200 images using D1000 Tapes and D1000 reagents.

**A**, Indexed samples were found to be in the expected size range (600-650 bp). **B**, Sizes corresponding to the bands detected in the ladder in the first lane of panel A.

**
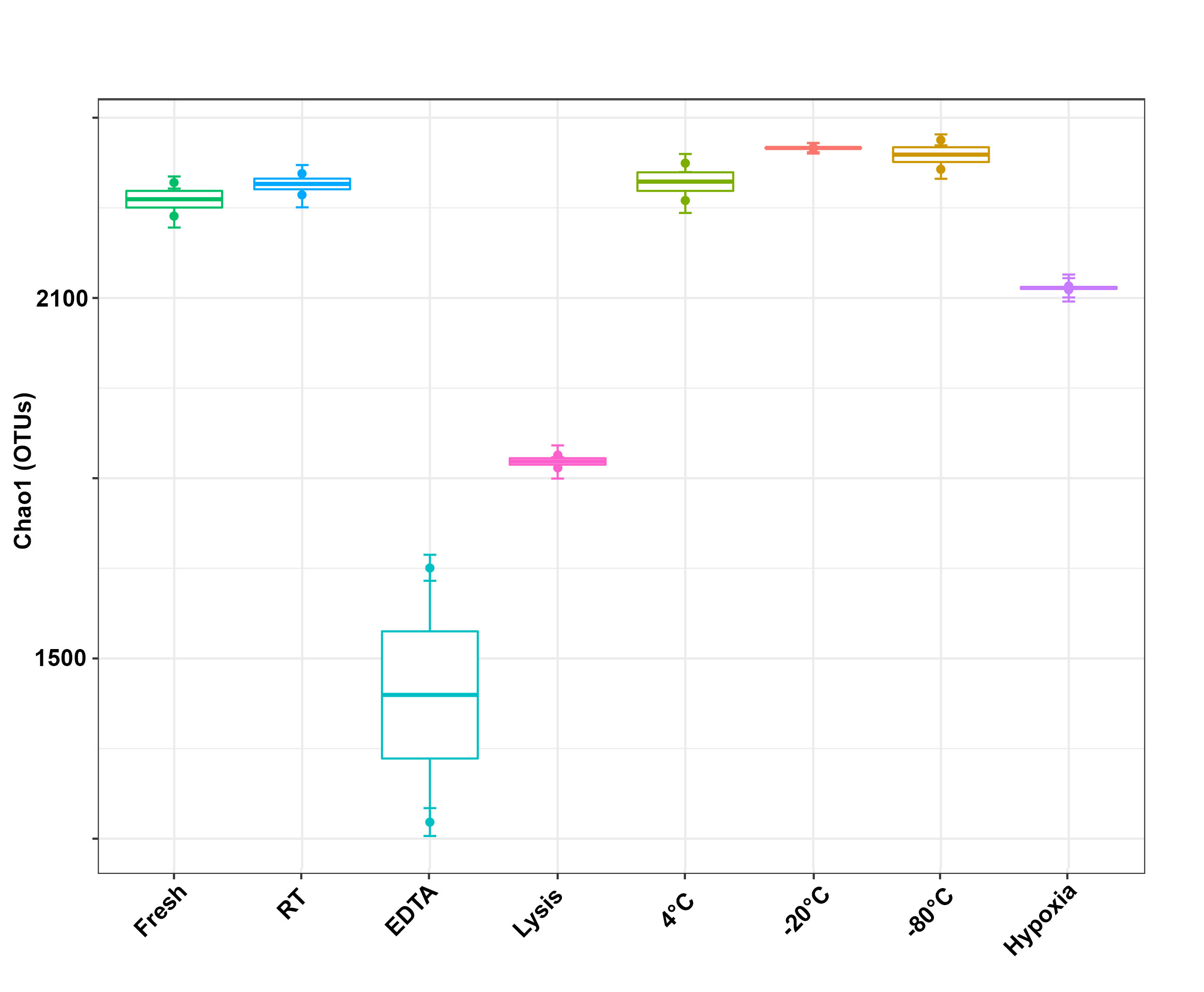
**

**
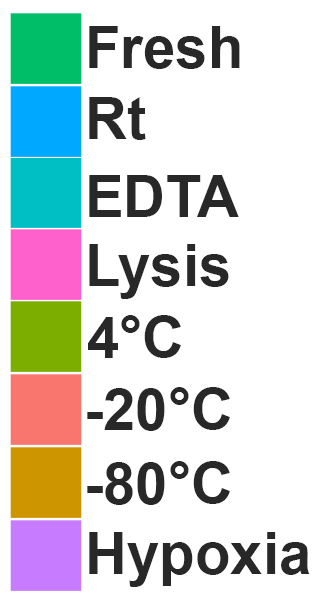
**

**Figure S2,** Detected alpha diversity in fecal matter following incubation under 8 different storage conditions. Chao1 richness estimator between storage conditions and individual samples.
